# Breast Cancer and Obesity: *In Vitro* Interferences between Adipokines and Proangiogenic Features and/or Antitumor Therapies?

**DOI:** 10.1371/journal.pone.0058541

**Published:** 2013-03-15

**Authors:** Virginie Dubois, Laetitia Delort, Hermine Billard, Marie-Paule Vasson, Florence Caldefie-Chezet

**Affiliations:** 1 Clermont Université, Université d’Auvergne, UFR Pharmacie, Laboratoire SVFp, Clermont-Ferrand, France; 2 INRA, UMR 1019, ECREIN, CRNH Auvergne, Clermont-Ferrand, France; 3 Centre Jean-Perrin, Unité de Nutrition, Clermont-Ferrand, France; 4 CLARA, Région Lyon Auvergne, Rhône-Alpes, France; University of South Alabama, United States of America

## Abstract

Obesity is now considered as a risk factor for breast cancer in postmenopausal women. Adipokine levels are modulated in obesity, and may play a role in carcinogenesis. Moreover, obesity increases risk of cancer mortality. Here, we hypothesized that this increase could be due to a modification in angiogenesis, capital event in the development of metastases, and/or in effectiveness of cancer treatments. To test these assumptions, following a same experimental design and simultaneously the effects of leptin and adiponectin on angiogenesis were investigated, and the impact of hyperleptinemia on anticancer drug effectiveness was measured in physiological and obesity situations. Focusing on angiogenesis, the proliferation of endothelial cells (HUVEC), which expressed leptin and adiponectin receptors, was stimulated by leptin and inhibited by adiponectin. Both adipokines globally reduced apoptosis and caspase activity. Leptin increased migration whereas adiponectin decreased migration, and leptin enhanced the area of the tubes formed by HUVEC cells while adiponectin inhibited their formation. MCF7 and MDA-MB-231 cells treated with leptin secreted more VEGF than untreated cells, whereas adiponectin treatment inhibited VEGF secretion. Finally, MCF7 cells pre-treated with leptin were more invasive than untreated cells. This effect was not reproduced in MDA-MB-231 cells. In the MCF7 breast cancer cell line, leptin could induce cell proliferation and reduced the efficacy of all breast cancer therapies (tamoxifen, 5-fluorouracil, taxol and vinblastin). These results suggest that, in obesity situation, leptin– in contrast to adiponectin – may promote tumor invasion and angiogenesis, leading to metastases ‘apparition, and reduce treatment efficacy, which could explain the increased risk of cancer mortality in cases of overweight. The evidence suggests adipokines influence breast cancer issue and could play a significant role, especially in obese patients for which hyperleptinemia, hypoadiponectinemia and increased metastatic potential are described.

## Introduction

Obesity, characterized as an excess of adipose tissue [1], is now a global health problem associated with a variety of metabolic diseases such as diabetes and an increased risk of developing cancer, including breast cancer [2], [3]. Furthermore, clinical studies indicate that increased BMI is associated with increased breast cancer recurrence, more aggressive tumors, and a shorter survival period [4]–[6].

The cellular and molecular mechanisms underlying the relationship between obesity and breast cancer are not fully understood. Adipose tissue is now widely considered as an active endocrine organ, and there is an increasing evidence to suggest adipose tissue may play an important role in breast cancer development by surrounding the mammary gland. A potential explanatory factor is the higher circulating estrogen levels attributed to elevated aromatase activity in larger adipose tissue depots [7]–[9]. Furthermore, the excess of fat increases the synthesis and secretion of bioactive peptides called adipokines, which include leptin and adiponectin [6], [10]–[13].

Leptin, the Ob gene product, is recognized as an adipocyte-secreted protein. It is a 16-kDA circulating hormone that, through the hypothalamus, plays a key role in satiety, energy expenditure and thermogenesis and is purportedly involved in controlling reproduction, hematopoiesis, and immunity [10], [14], [15]. Leptin expression and plasma concentrations were found to be markedly increased in obese people and positively correlated to body fat mass [16], [17].

Adiponectin is a 224-amino-acid circulating adipokine [16] that is implicated in metabolic and vascular regulation. There is recent evidence that adiponectin may protect against diabetes and arteriosclerosis [18], [19]. It is the most abundant protein hormone synthesized by adipose tissue, and is strongly down-regulated in obesity [16], [20].

*In vitro* assays studying the effects of leptin and adiponectin on cell proliferation have found a stimulatory role of leptin and an inhibitory role of adiponectin in mammary tumor growth [27]–[29]. These observations suggest that leptin and adiponectin exert procarcinogenic and anticarcinogenic effects, respectively.

The main hypothesis of this study is that these adipokines may affect prognosis of breast cancer for overweight or obese patients. Indeed, clinical studies correlate high leptin levels [17], [21]–[23] and low adiponectin levels in women to an increased risk of breast cancer [11], [17], [22], [23], metastatic tumor phenotype [24] with poor prognosis [25], [26]. This increase in cancer-related mortality could be explained by two complementary possibilities: an increase of metastasis development and/or a decrease in therapy effectiveness in overweight and obese patients.

The first argument is based on the fact that obesity could increase the risk of metastasis in breast cancer through the process of angiogenesis and tumor invasion. Angiogenesis, the formation and organization of new blood vessels from pre-existing vessels, contributes to both physiological and pathological events [30], [31]. Angiogenesis encompasses a cascade of processes involving the proliferation, migration and remodeling of endothelial cells in response to growth factors and cytokines. This angiogenic process requires proangiogenic factors together with vascular endothelial growth factor (VEGF), fibroblast growth factor (FGF) and anti-angiogenic agents such as endostatin and angiostatin [30], [31]. We thus hypothesize that adipocytes *via* locally secreted adipokines might be active regulators of this process.

Many studies have shown that adipocytes are not just energy storage sites but also important sources of cytokines such as tumor necrosis factor-α and VEGF [15], [31]–[34], a number of which are potentially implicated in regulating angiogenesis [19]. One study strategy is to focus on the adipocyte microenvironment of the mammary gland and the potential effects, *via* soluble adipokines, on decreasing or increasing proliferative, invasive and angiogenic activities of ductal epithelial cells [35], [36].

The second argument relies on a decrease of therapy effectiveness. Indeed, we found recently a close relationship between estrogen and the leptin pathway and a stimulatory effect of leptin on the *in vitro* tumor expression of aromatase [29], [37] and these results suggest leptin could adverse effects on the efficacy of breast cancer treatment, particularly anti-estrogen treatments such as tamoxifen.

However, this has prompted several studies on the relationship between leptin and anticancer treatment. Tamoxifen (Tx) treatment reportedly increases serum leptin in patients with postmenopausal breast cancer, regardless of stage of disease [38]. It is possible that anti-estrogen treatment could stimulate the expression of estrogen receptors which, in turn, would regulate adipocyte leptin secretion [39]. However, another study with tamoxifen showed that it had no effect on serum leptin levels [40]. Moreover, leptin has been shown to interfere with non-hormonal therapies, since the *in vitro* cytotoxic effect of 5-fluorouracil (5 FU) on colon cancer cells is diminished in presence of leptin [41].

The objective of this study was to highlight the relationship between obesity and increase risk of mortality in breast cancer. For this, we investigated the relationship between adipocyte secretions (adipokines) and features of angiogenesis or effectiveness of anti-tumor therapies. To explain that, two additional assumptions were made and tested: leptin and/or adiponectin could be able to modify, by a systemic effect, angiogenesis events and hyperleptinemia could affect cancer treatment effectiveness in obesity situation.

For this purpose, the impact of adipokines on the tumor invasion and angiogenesis process has, for the first time, been characterized by comparing leptin and adiponectin used at doses mimicking plasmatic levels of leptin and adiponectin similar to those of healthy or obese conditions. Indeed, a best understand of potential mechanisms by which adipokines modulate angiogenesis and apoptosis may permit to infer on the potential consequences in obese or overweight people. Moreover, we went on to investigate the effects of interactions between leptin and anticancer therapies such as tamoxifen (Tx), 5-fluorouracil (5 FU), taxol and vinblastin, on MCF7 breast cancer cell proliferation.

## Materials and Methods

### Materials

RPMI 1640, Leibovitz’s L-15 medium, L-glutamine, gentamycin and trypsin were purchased from Pan Biotech Gmbh (Aidenbach, Germany). EndoGRO-VEGF complete media was purchased from Millipore (Molsheim, France). Fetal bovine serum and epidermal growth factor (EGF) was from Abcys S.A. (Paris, France). Trizol was obtained from Invitrogen (Carlsbad, USA). Human recombinant leptin was provided by R&D (Abingdon, UK), and human recombinant adiponectin was obtained as previously described (Neumeier et al. 2006). Anticancer treatments were from Sigma (Saint-Quentin Fallavier, France).

### Cell Culture

Human umbilical primary vein endothelial cells (HUVEC, pool of 3 donors) from Millipore (Molsheim, France) and isolated as previously described (42) were cultured in EndoGRO-VEGF complete media containing 2% fetal calf serum (FCS), rhVEGF (5 ng/ml), EGF (5 ng/ml), bFGF (5 ng/ml), IGF1 (15 ng/ml), ascorbic acid (50 µg/ml), hydrocortisone (1 µg/ml), heparin sulfate (0.75 U/ml) and glutamine (10 mM). This study used HUVEC subcultures from passages 2 to 8.

Two human mammary breast cancer cell lines differing in terms of estrogen receptor (ER) status were chosen for this study, i.e. the ER-positive MCF7 line and the ER-negative MDA-MB-231 line, both obtained from the American Type Culture Collection. These cells were routinely grown in a phenol-red-free RPMI 1640 and Leibovitz’s L-15 medium, respectively, supplemented with 10 to 15% heat-inactivated FCS, L-glutamine (2 mM) and gentamicin (50 µg/ml) at 37°C, at under 5% or without CO_2_ atmosphere, respectively. This study used MCF7 cultures from passages 35 to 40.

Leptin and adiponectin were dissolved in phosphate-buffered saline. For all data shown, each individual experiment represented by *n* values was performed with independently-prepared cells.

The presence of leptin and/or adiponectin was search in serum and culture media by ELISA (Human Leptin ELISA Kit, RayBiotech, USA) and the result was negative.

### HUVEC

- Expression of mRNA of Ob-R, AdipoR1 and AdipoR2: To determine whether endothelial cells express leptin and adiponectin receptors, PCR analysis was performed on RNA extracts from cultured cells. Total RNA from cultured cells was isolated with Trizol according to the manufacturer’s protocol and quantified using a Nanodrop spectrophotometer (Nanodrop 2000, Thermo scientific, Waltham, USA).

Reverse transcription was conducted from 2 µg of total RNA, yielding 20 µl of cDNA, using the “High-Capacity cDNA Reverse Transcription Kit” (Applied Biosystems, Courtaboeuf, France).

Rt-synthesized cDNA were amplified using primers 5′-CATTTTATCCCCATTGAGAAGTA-3′ and 5′-CTGAAAATTAAGTCCTTGTGCCCAG-3′. q-RT-PCR was realized using Sybergreen following the manufacturer’s instructions on a StepOne system (Applied Biosystems, Courtaboeuf, France).

- Expression of protein of Ob-R, AdipoR1 and AdipoR2: The expression of Ob-R and AdipoR was investigated by immunohistochemical staining using affinity-purified goat polyclonal biotinylated antibodies against Ob-R and AdipoR (R&D, Abingdon, UK).

Cells were grown on plastic slides for 48 h in a 37°C humidified atmosphere with 5% CO2 before fixing with acetone for 10 min. Non-specific binding sites were blocked using the avidin/biotin kit for 30 min (Abcys, Paris, France). Slides were then incubated overnight at 4°C in a humidified chamber with the anti-Ob-R and anti-AdipoR biotinylated antibodies (1 µg/ml). Endogenous peroxidase activity was inhibited with 0.3% hydrogen peroxide for 5 min. Visualisation was carried out using a Vectastain ABC peroxidase-conjugated streptavidine kit for 30 min (Abcys, Paris, France). The sections were then treated with DAB substrate for 10 min to give staining. Finally, slides were contrasted using haematoxylin, dehydrated and mounted using the Vectastain mounting medium (Abcys, Paris, France). For each assay, control samples without the anti-Ob-Rt or anti-adipoR antibody or without the peroxidase revelation kit were used to establish the specificity of the immunohistochemical analysis.

- VEGF secretion: VEGF expression by HUVEC after stimulation by leptin (10 to 1 000 ng/ml) or adiponectin (1 000 to 10 000 ng/ml) was measured on cultured media *via* ELISA according to the manufacturer’s instructions (Raybiotech, Norcross, CA, USA), with spectrophotometric readings at 450 nm. The minimum detectable dose of VEGF was typically less than 20 pg/ml, with over 90% intra-assay reproducibility and over 88% inter-assay reproducibility. The assay was performed once in duplicate wells for each concentration tested.
- Proliferation assays: Cells were plated (5×10^3^ cells) in 96-well plates in medium without VEGF and bFGF for leptin experiments and in complete medium for adiponectin. After 24 h, cells were exposed to fresh medium without VEGF and bFGF, containing different concentrations of human leptin reflecting serum leptin concentrations within normal physiological concentration (10 ng/ml or 0.625 nmol/l), in case of obesity (100 ng/ml or 6.25 nmol/l), and at pharmacological level (1,000 ng/ml or 62.5 nmol/l), or in complete fresh medium with human adiponectin at the concentrations of 100 or 1,000 ng/ml (concentrations found in obesity) and 10,000 ng/ml (within normal physiological concentration). After 72 and 96 h, the medium was removed and recovered by 200 µl of a 25 µg/ml solution of resazurin in PBS with 10% FCS. The plates were incubated for 2 h at 37°C in a humidified atmosphere containing 5% CO_2_. Fluorescence was measured on an automated 96-well plate reader (Fluoroskan Ascent FL, Thermo Fisher Scientific, Wilmington, USA) using an excitation wavelength of 530 nm and an emission wavelength of 590 nm. The cell proliferation assay was performed at least 4 times in replicates of at least 3 wells for each concentration tested. Results were expressed as percentage growth compared to control at a given timepoint.
- Apoptosis analysis:

Global apoptosis: Apoptosis was evaluated using the Annexin V-FITC kit (Beckman). Cells were plated (165,000 cells) in 6-well plates in complete medium. After 24 h, the cells were exposed to fresh medium without VEGF and bFGF, containing different concentrations of human leptin (10 ng/ml, 100 ng/ml and 1 000 ng/ml) or complete fresh medium with human adiponectin (100, 1 000 and 10 000 ng/ml). After 72 h of treatment, the cells were harvested with trypsin, and 10% FCS in PBS was added as soon as the cells were removed from the dish. Then, 3×10^5^ cells were washed with PBS and incubated for 10 min with Annexin V-FITC and propidium iodide (PI) following the manufacturer’s protocol. Cells were analyzed on a cytometer (Beckman), placing the FITC signal in FL1 and the PI signal in FL4. Intact cells were gated in the FSC/SSC plot to exclude small remains. Cells in the lower-right quadrant of the FL1/FL4 dot plot (labeled with Annexin V-FITC only) were considered to be in early apoptosis, and cells in the upper-right quadrant (labeled with Annexin V-FITC and PI) were considered to be in late apoptosis/necrosis. The apoptosis assay was performed at least in triplicate for each concentration tested.

Caspase assay: Caspase activation (caspases 2, 3, 8 and 9) in HUVEC after stimulation by leptin (10 to 1 000 ng/ml) or adiponectin (1 000 to 10 000 ng/ml) for 72 h was measured on ApoAlert Caspase Assay Plates® following the manufacturer’s protocol (Clontech, Montain View, CA, USA) with a spectrophotometric reader (380 nm excitation filter and 460 nm emission filter). The assay was performed once in duplicate wells for each concentration tested.

- Migration assay: The migration of HUVEC was assayed by the wound healing method [42]. Cells (80,000 cells per well) were seeded into a 24-well plate and incubated with complete medium (37°C, 5% CO_2_). At full confluence, cells in each well were scraped away horizontally using a 200 µl tip. At this moment, randomly selected views along the scraped line were measured and photographed using a microscope with a camera at 40× magnification and used to mark the beginning of the migration assay. The medium was then replaced by fresh VEGF-free medium with human leptin at 10, 100 and 1 000 ng/ml or complete fresh medium with human adiponectin at 100, 1 000 and 10 000 ng/ml.

After 22 h of incubation, pictures of the selected views were taken for a second time in order to measure the reduction in the scraped area, corresponding to cell migration. The change in area of the experimental condition was compared against the control. This assay was performed at least 6 times. Results were expressed as mean normalized relative to the control.

- Endothelial tube formation: The ability of adipokines to modify endothelial cell capillary tube formation was tested here using a Matrigel® model. When HUVEC are cultured on Matrigel®, a solid gel of mouse basement membrane proteins, they normally form tube-like structures. In brief, using a 96-well plate, 50 µl of Matrigel® (BDBiosciences, California, USA) (9.5 mg/mL) held at 4°C was transferred into each well. Once the Matrigel® had solidified (1 hour, 37°C, 5% CO_2_), HUVEC (10 000 cells) were placed on top of the matrix along with complete medium supplemented with human leptin at 10, 100 and 1 000 ng/ml or human adiponectin at 100, 1 000 and 10 000 ng/ml. After 24 h, endothelial tube formation was observed in full each well under a microscope (×40, Olympus, France) with a camera (Astra 20, Soft imaging system) and analyzed with ImageJ software (National Institutes of Health, Bethesda, MD, USA) [43]. A custom made plugin was specially developed by Pr Birembaut, at INSERM UMRS 903, Reims, France (data not published). Assays were performed at least 5 times. Results are expressed in numbers of formed tubes and middle areas.

### MCF7 and MDA-MB-231 Cells

- VEGF secretion: The expression of VEGF by MCF7 and MDA-MB-231 cells after stimulation by leptin (10 to 1,000 ng/mL) was measured as previously described for HUVEC cells.
- Migration assay: The migration of MCF7 and MDA-MB-231 cells was assayed after treatment by leptin (10 to 1,000 ng/mL) as previously described for HUVEC.
- Invasion assay : A two-level 24-well cell migration assay chamber was used for these experiments. A membrane pre-coated with 100 µl of Matrigel® (25 µg) was placed on top of the bottom chamber (4 h, 37°C) and dried overnight at room temperature before being rehydrated with medium without serum. Then, in the top chamber, 1.5 × 10^5^ MCF-7 or MDA-MB-231 cells pre-treated for 48 h with leptin (10; 100 and 1 000 ng/ml) were transferred to each of the wells. Migration was evaluated by addition of serum (10%) to the lower compartment for 48 hours. The membrane was washed and removed, and the cells were fixed with methanol for 10 min. Finally, the fixed cells were incubated in DAPI (1 µg/ml, 10 min), and the number of cells that had migrated through the membrane was photographed under a fluorescence microscope and counted using ImageJ software. These experiments were performed at least in triplicate. Results were expressed using an index of invasion.
- Proliferation assays with anticancer treatment: MCF7 breast cancer cells were plated (5 × 10^3^ cells) in 96-well plates and treated or not with leptin used at doses reflecting serum concentrations within normal physiological concentration (10 ng/ml), obesity level (100 ng/ml) and pharmacological level (1 000 ng/ml), in presence or not of Tx (0.75 µM), 5-FU (0.75 µM), taxol (1 nM) or vinblastin (1 nM). Cell proliferation was detected using fluorescent resazurin (25 µg/ml, 590 nm) at different timepoints (24, 48, 72 and 96 h post-incubation) using a microplate reader (Fluoroskan Ascent FL®) at an excitation wavelength of 530 nm and an emission wavelength of 590 nm. Under these conditions, fluorescence was proportional to number of living cells [29]. The cell proliferation assay was performed 6 times in replicates of 6 wells for each concentration tested. Results are expressed as percentage growth compared to control at a given timepoint.

### Statistics

Statistical analysis was performed using the paired, bilateral Student’s *t*-test with StatView Software version 5.0 (SAS Institute Inc.). Differences at p<0.05 (flagged as *) were considered statistically significant.

## Results

### Impact of Leptin and Adiponectin on Angiogenesis Using HUVEC

#### Expression of Ob-R, AdipoR1 and AdipoR2

**Figure 1:**
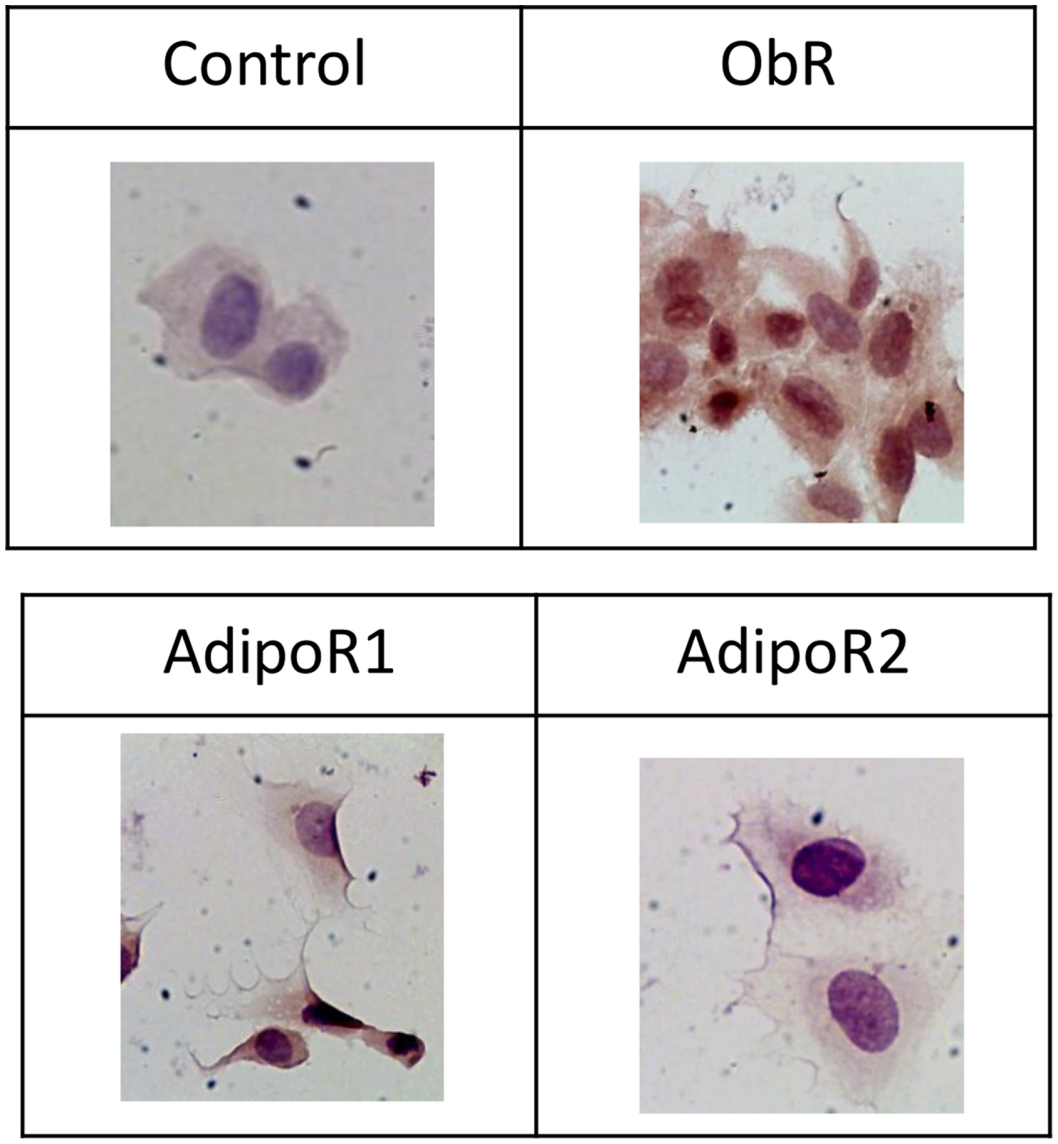
Protein expression of leptin and adiponectin receptors in untreated HUVEC. The protein expression was analysed by immunochemistry (magnification x 400).

Ob-R, AdipoR1 and AdipoR2 mRNA (data not shown) and protein (Fig. 1) were found to be expressed in HUVEC.

#### VEGF expression

VEGF concentrations in HUVEC after 72-h stimulation with leptin (10 to 1 000 ng/ml) or adiponectin (100 to 10 000 ng/mL) were measured. No secretion of VEGF was found in the HUVEC supernatant (data not shown).

#### Proliferation assays

**Figure 2:**
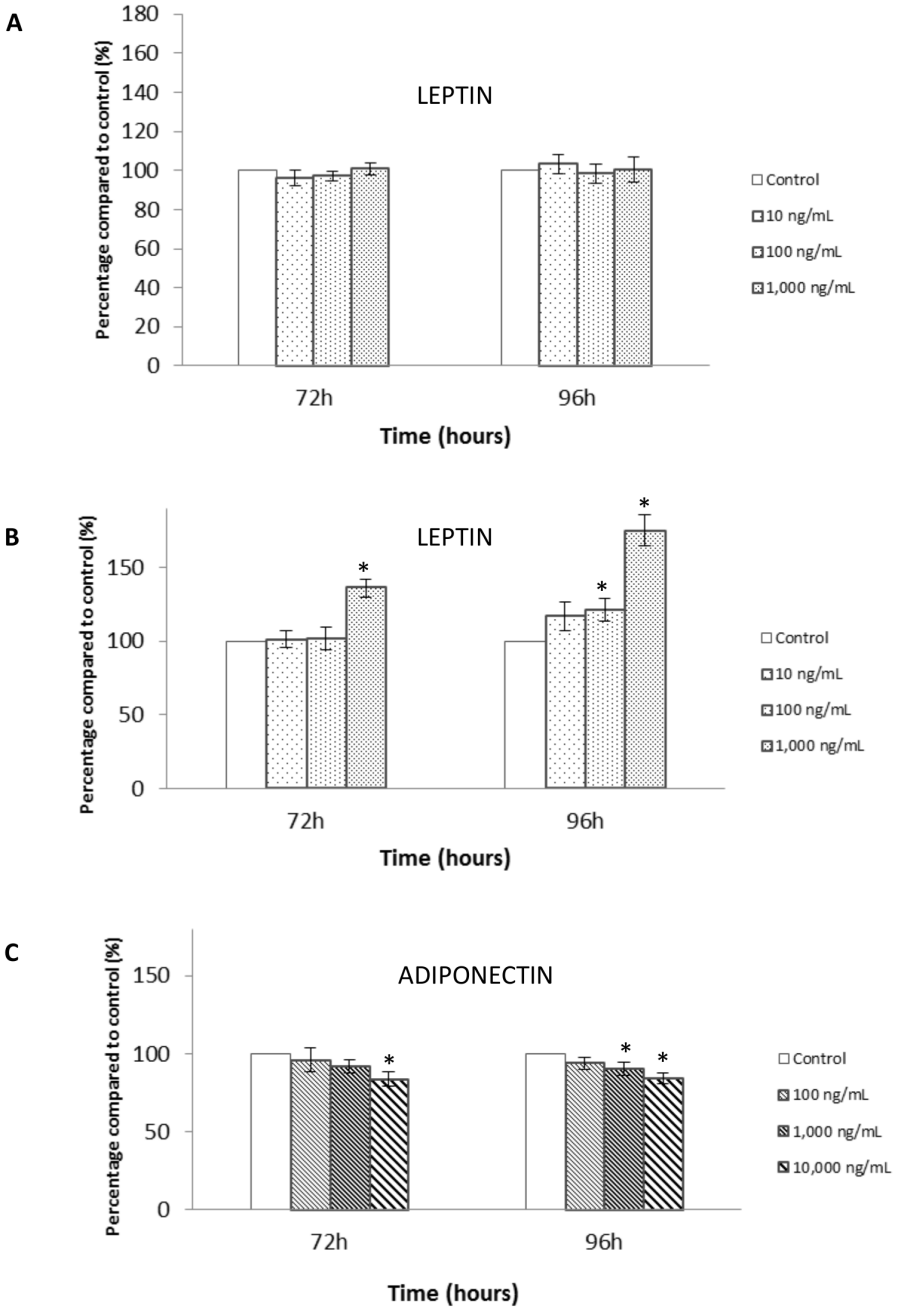
Effect of leptin or adiponectin on the proliferation of HUVEC cells. **A.** HUVECs were cultured in the presence of increasing concentrations of human recombinant leptin (10 to 1,000 ng/mL) for 72 and 96 hours in a complete medium containing VEGF and bFGF (n = 6). **B.** HUVECs were cultured in the presence of increasing concentrations of human recombinant leptin (10 to 1,000 ng/mL) for 72 and 96 hours in a medium without VEGF and bFGF (n = 6). **C.** HUVECs were cultured in the presence of increasing concentrations of human recombinant adiponectin (100 to 10,000 ng/mL) for 72 and 96 hours in a complete medium with VEGF and bFGF (n = 4). Data are presented as mean ± SEM *p<0.05 *vs* control.

In complete medium containing VEGF and bFGF, human leptin had no effect on HUVEC proliferation (Fig. 2A). However, when the experiment was realized without VEGF and bFGF, human leptin stimulated HUVEC proliferation in a dose-dependent manner, and a significant increasing effect was observed at concentrations reflecting obesity conditions and pharmacological concentrations at 96 h (+21 and +75% at 100 and 1 000 ng/mL respectively, p<0.05) compared to leptin-free controls (Fig. 2B).

Adding normal physiological concentrations of human adiponectin (10 000 ng/ml) to HUVEC resulted in significant inhibition of endothelial cell proliferation at 96 h (−15%, p<0.05) compared to adiponectin-free controls (Fig. 2C). The adiponectin concentration corresponding to obesity (1 000 ng/ml) also decreased cell growth at 96 h (−10%, p<0.05). These effects can maybe be explained by a change of apoptosis.

### Apoptosis Assays

**Figure 3:**
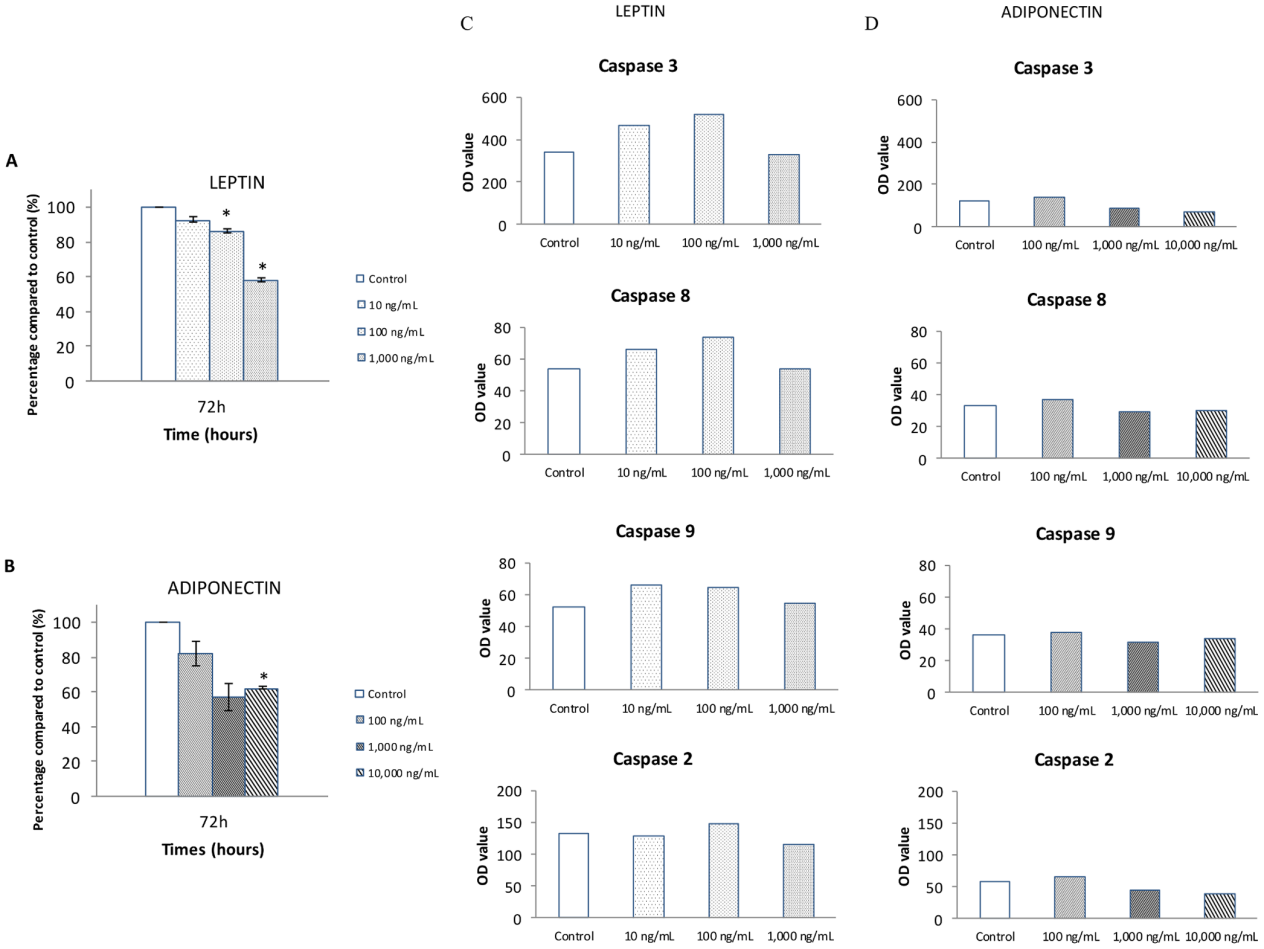
Effect of leptin and adiponectin on global apoptosis and on caspase activation in HUVEC cells. **A.** HUVECs were cultured in the presence of increasing concentrations of human recombinant leptin (10 to 1,000 ng/mL) for 72 in a medium without VEGF and bFGF (n = 3). **B.** HUVECs were cultured in the presence of increasing concentrations of human recombinant adiponectin (100 to 10,000 ng/mL) for 72 hours in a complete medium (n = 3). Data are presented as mean ± SEM *p<0.05 vs control. **C.** HUVECs were cultured in the presence of increasing concentrations of human recombinant leptin (10 to 1,000 ng/mL) for 72 in a medium without VEGF and bFGF (n = 1) **D.** HUVECs were cultured in the presence of increasing concentrations of human recombinant adiponectin (100 to 10,000 ng/mL) for 72 hours in a complete medium (n = 1).

- Global apoptosis: After 72 h of treatment with recombinant human leptin at 10, 100 and 1 000 ng/mL, the population of HUVEC in apoptotic stage decreased by 8, 14 and 42%, respectively (Fig. 3A).

Adiponectin at 10 000 ng/ml decreased apoptosis of HUVEC by 38% (Fig. 3B).

- Caspases assay: To investigate apoptotic pathways, the activation of endothelial caspases was analyzed after 72 h of treatment. Leptin at 10 and 100 ng/ml and adiponectin at 100 ng/ml increased caspase activation, whereas leptin at 1 000 ng/mL and adiponectin at 1 000 and 10 000 ng/ml decreased caspase activity (Fig. 3C).

#### Migration assays

**Figure 4:**
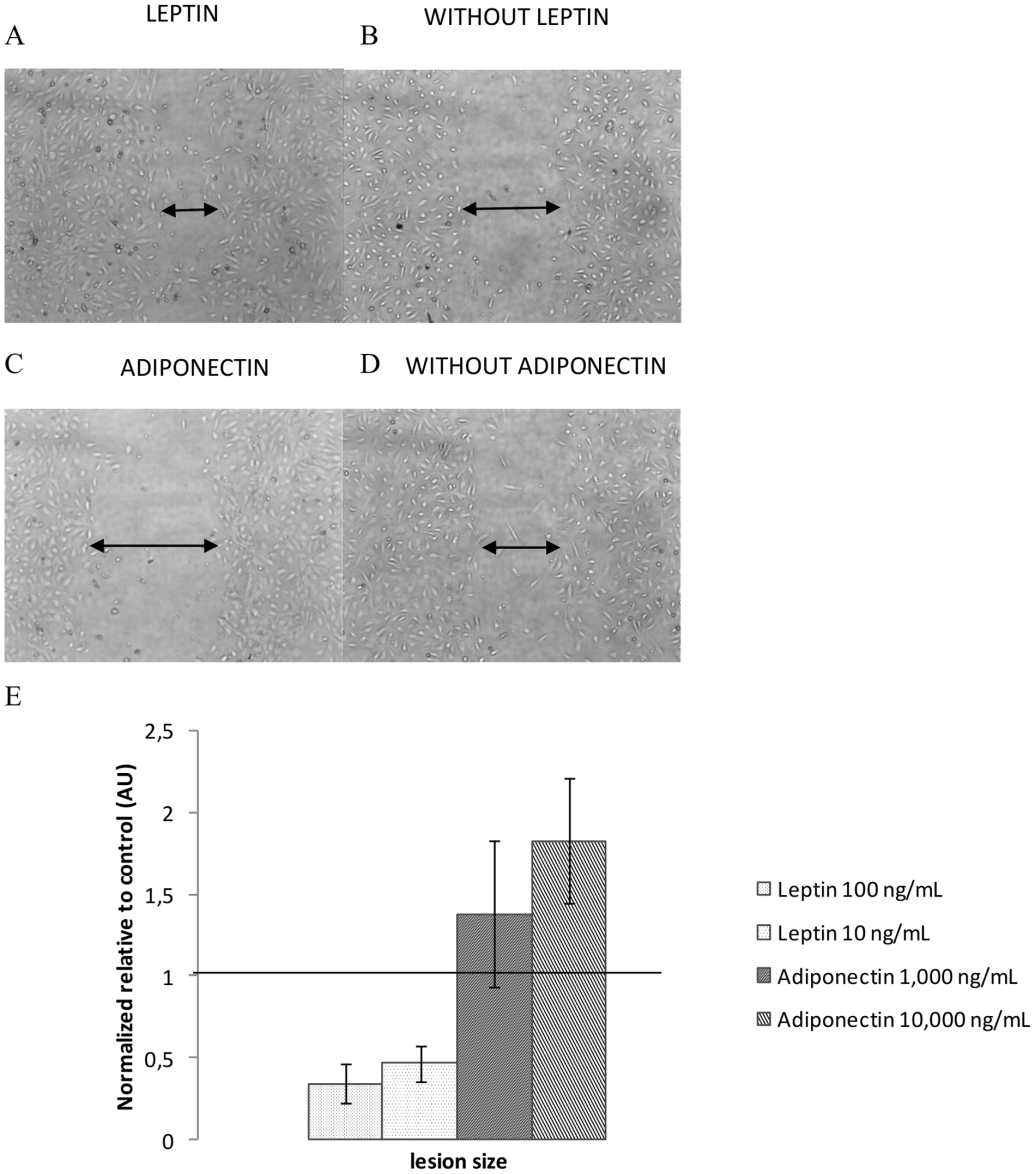
Migration assay for HUVECs treated by leptin or adiponectin. Migration pattern of HUVECs with (**A**) or without (**B**) leptin treatment (100 ng/mL) and with (**C**) or without (**D**) adiponectin traitement (10,000 ng/mL). The percentage of migration ability (**E**) was analyzed by averaging the scraped area of each well under each condition (n = 6). Original magnification ×40 (A, B, C and D). Data were expressed as mean ± SEM (E and F). *p<0.05 vs control.

We determined the effect of adipokines on endothelial cell migration using the wound-healing method. At 22 h post-wounding of HUVEC, little migration was measured in controls, whereas leptin triggered a strong, dose-dependent increase in migration (Fig. 4). Compared with controls, leptin at the concentration of 100 ng/ml increased cell migration by +66% (p<0.05 *vs* control).

Conversely, normal physiological adiponectin concentrations actually inhibited HUVEC migration (−82%, p<0.05 *vs* control), whereas concentrations reflecting obesity had no effect.

#### Evaluation of endothelial tube formation

**Figure 5:**
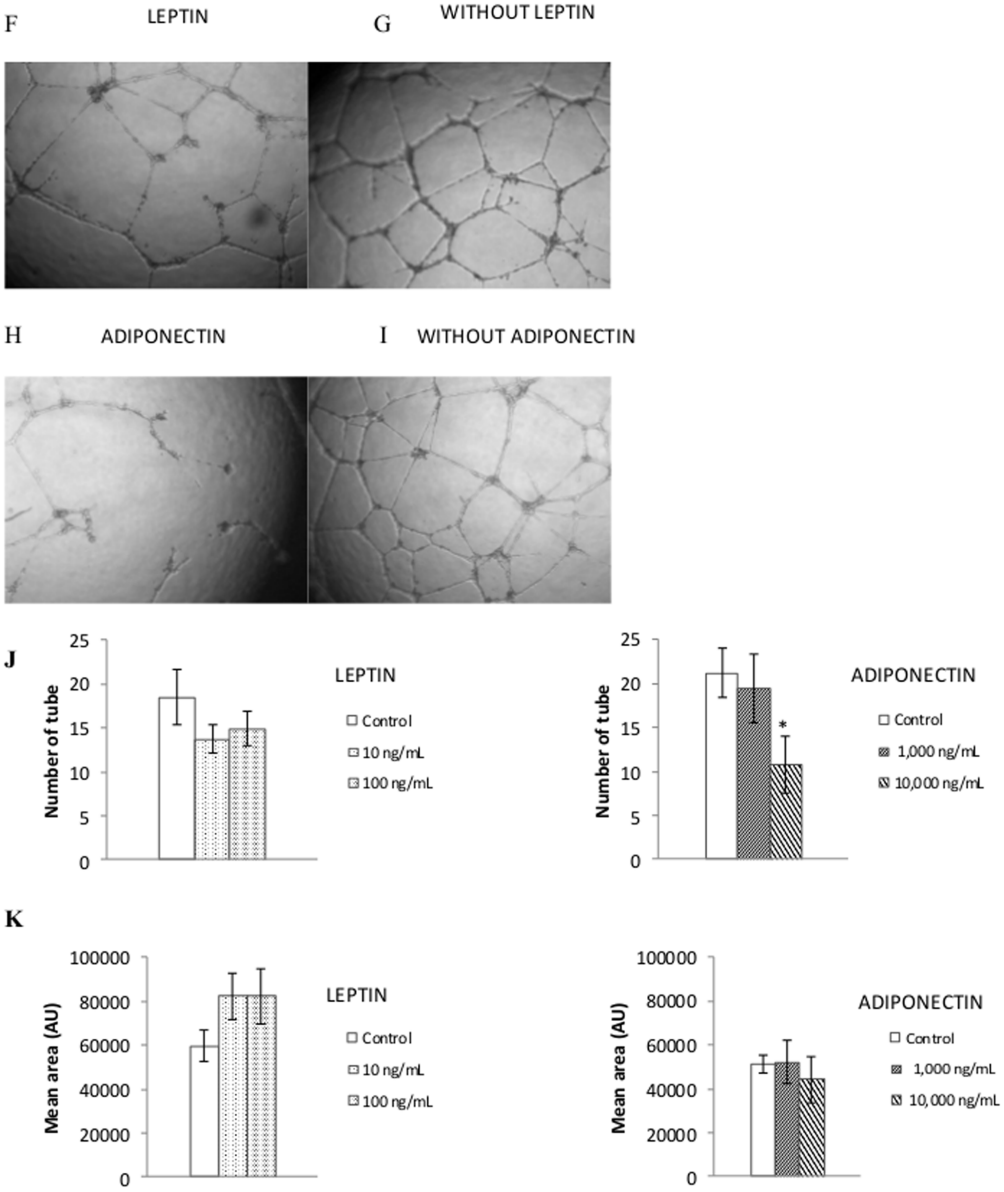
Endothelial tube formation for HUVECS treated by leptin or adiponectin. Differentiation of HUVEC in three-dimensional Matrigel cultured in treatment with (**A**) or without (**B**) leptin (100 ng/ml). Differentiation of HUVEC in three-dimensional Matrigel cultured in treatment with (**C**) or without (**D**) adiponectin (10,000 ng/ml). Average of number of tube (**E**) and mean area (**F**) in different concentrations of leptin or adiponectin-treated HUVEC was calculated using computer software (ImageJ software) (n = 6). Original magnification ×40 (A, B, C and D). Data were expressed as mean ± SEM (E and F). *p<0.05 vs control.

As shown in Figure 5, exposed HUVEC cultured on Matrigel® to leptin (10 to 100 ng/ml) induced the formation of more elongated and bifurcating tubes compared to controls. Conversely, HUVEC exposed to adiponectin (10 000 ng/ml) failed to form the capillary structure. Quantitative measurements confirmed these results (Fig. 5). Indeed, leptin at 100 ng/ml led to a 20% decrease in the number of tubes formed but increased average tube area by 38%, whereas adiponectin at 10 000 ng/ml led to a 10% decrease in the number of tubes formed but did not alter their mean area (Fig. 5).

### Impact of Leptin on Angiogenesis and Invasion of Breast Cancer Cells

#### VEGF expression

**Figure 6:**
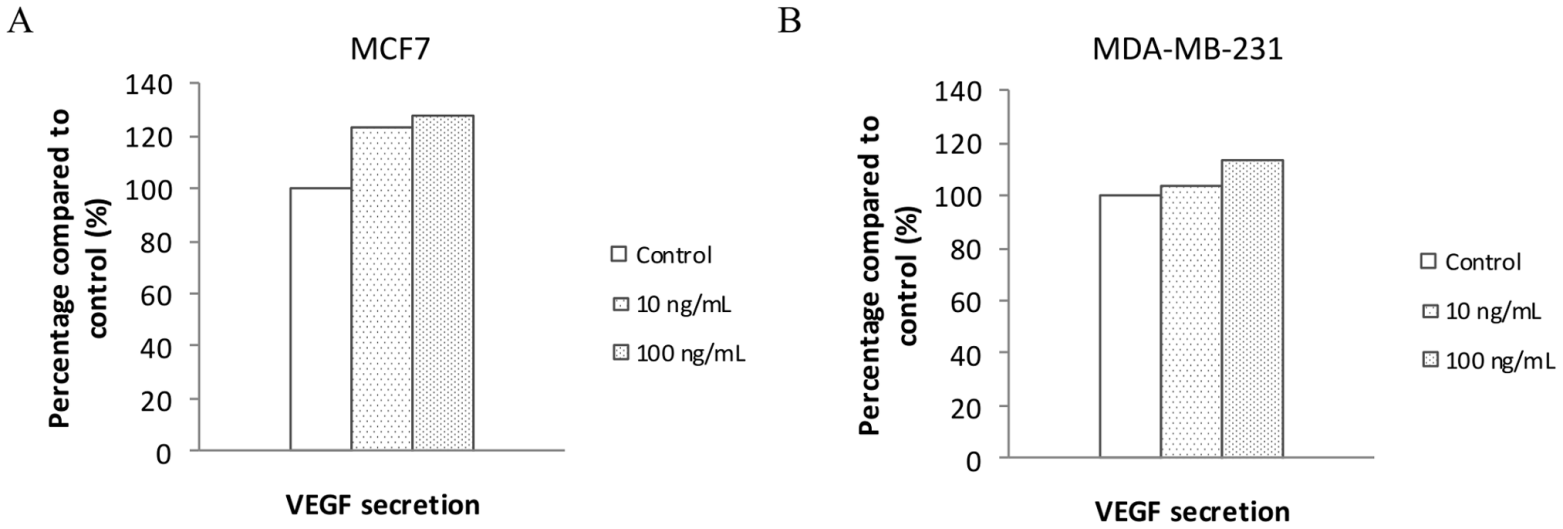
Effects of leptin on VEGF secretion in MCF7 (A) and MDA-MB-231 (B) (n = 1).

VEGF concentrations were measured in MCF7 and MDA-MB-231 mammary tumor cells after 72-h stimulation with leptin (10 to 1 000 ng/ml). Leptin at levels corresponding to obesity (100 ng/ml) and pharmacological concentrations (1 000 ng/ml) enhanced VEGF expression in MCF7 (Fig. 6A) and MDA-MB-231 cells (Fig. 6B).

#### Migration assays

Compared with controls, leptin at the concentration of 100 ng/ml increased MCF7 and MDA-MB-231 cell migration (by 6% and 22%, respectively; p<0.05 *vs* control) (data not shown).

#### Invasion assay on breast cancer cells

**Figure 7:**
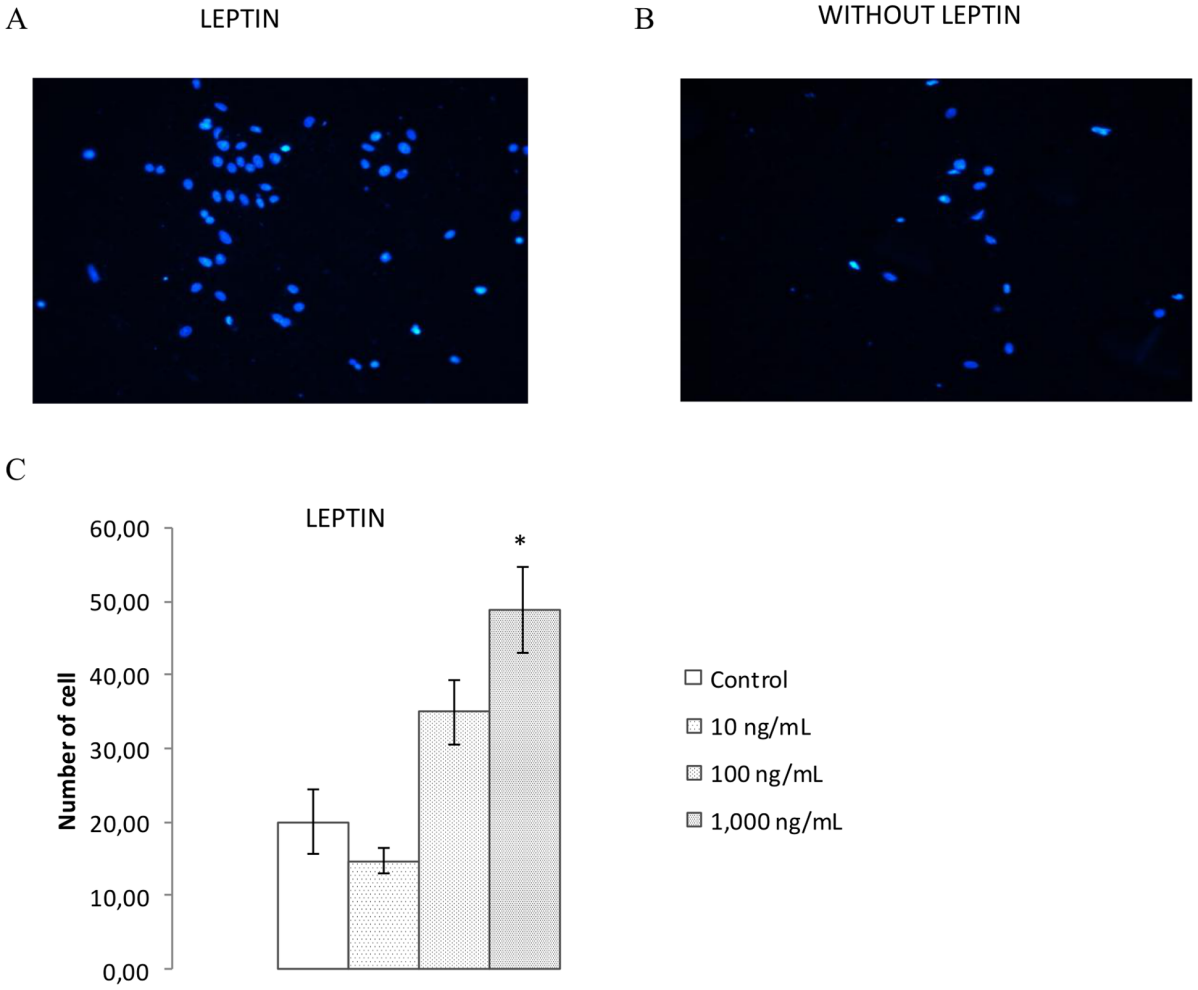
Effect of leptin on invasion of MCF7 cells. Invasion of MCF7 with (**A**) or without (**B**) leptin treatment (1,000 ng/mL). The number of cells (**C**) was analyzed under each condition (n = 3). Data expressed as mean ± SEM (C). *p<0.05 vs control.

As shown in Figure 7, MCF7 cells pre-treated with leptin at 1 000 ng/mL were more invasive than control cells. MDA-MB-231 cells showed no effect of leptin on their invasion process (data not shown).

### Impact of Leptin on Carcinolytic Drug Effects on the MCF7 Breast Cancer Cell Line

**Figure 8:**
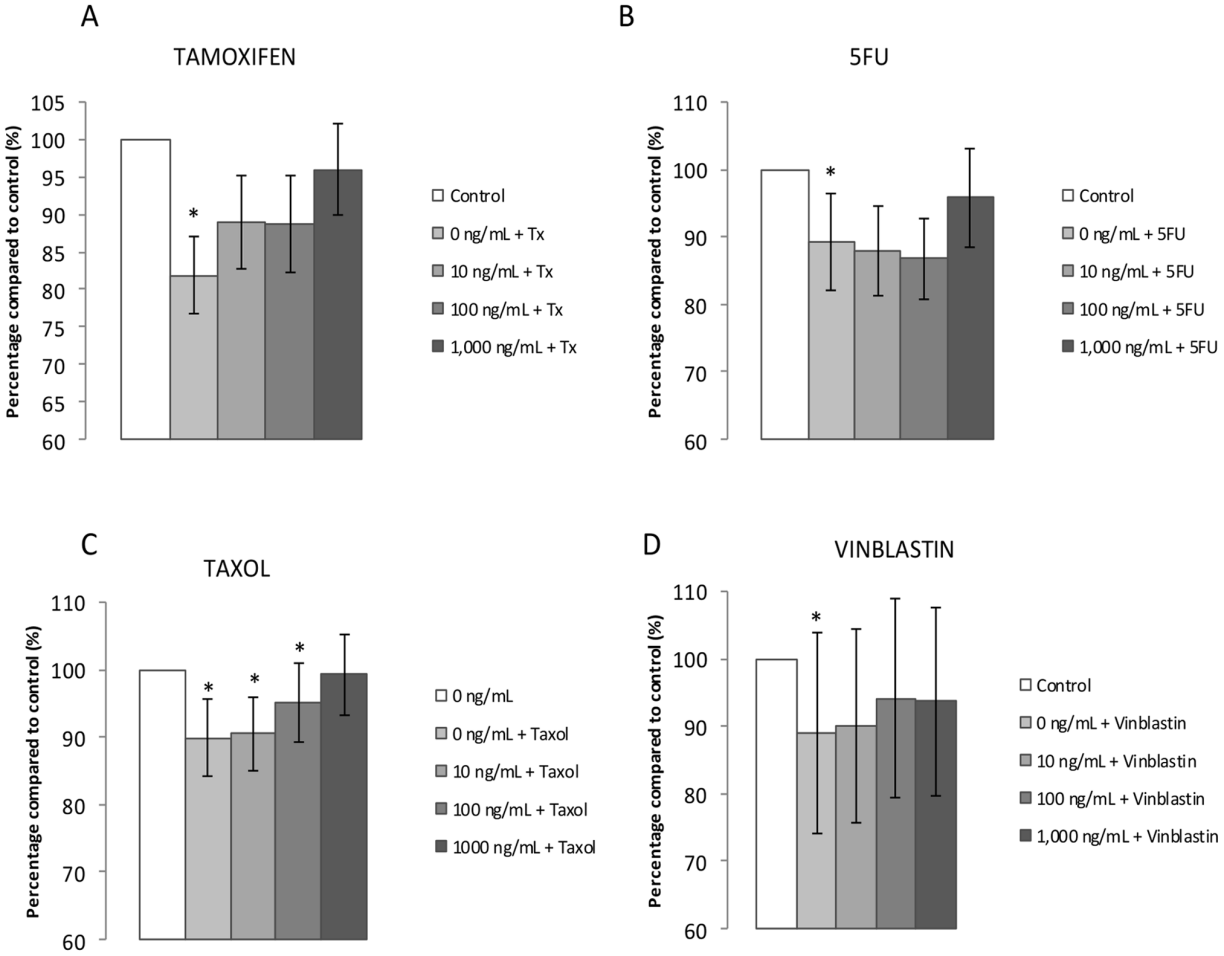
Effects of leptin and Tx (A), 5-FU (B), Taxol (C) and Vinblastin (D) on MCF7 cells proliferation at 72 h (n = 6). Data are presented as means ± SEM *p<0.05 vs control.

As previously reported, leptin at 100 and 1 000 ng/ml induces a significant increase in cell proliferation after 72 h (+15%) in the MCF7 cell line, but has no effect on MDA-MB-231 cells, so only MCF-7 cells were used for the following experiments [29]. Based on these results, we decided to use anticancer drug concentrations that inhibit cell proliferation by 10–20% at 72 h. After screening to determine the optimal concentration to use, the concentrations selected were 0.75 µM for Tx and 5-FU and 1 nM for taxol and vinblastin. Under these experimental conditions, cancer cell growth was significantly inhibited by Tx (−18%), 5-FU (−11%), taxol (−11%) and vinblastin (−11%) when used alone, at 72 h (data not shown). The presence of leptin at 10, 100 and 1 000 ng/ml led to a decreased inhibition of cell proliferation by Tx (−11%, −12% and −4% respectively), 5-FU (−13%, −14% and −5% respectively), taxol (−10, −5% and −1% respectively) and vinblastin (−10%, −6% and −7% respectively) at 72 h (Fig. 8). Thus, in the presence of leptin, the inhibition of cell proliferation by all anticancer therapies was reduced.

## Discussion

Epidemiological studies point to obesity as a risk factor for breast cancer and increased risk of mortality. We previously reported that the adipokines leptin and adiponectin were expressed in breast cancer tissue and had proliferative (leptin) and anti-proliferative (adiponectin) effects on breast cancer cell lines [29]. Here, we hypothesized that this increased mortality could be due to a change in metastasis and/or in the efficiency of cancer treatments. The present setting, using model on tumour sites distant from the adipose tissue, might try to partially reconstruct the microenvironment of tumours in presence of plasmatic levels of leptin and adiponectin similar to those of overweight or obese patients. These adipokine levels are probably quite diluted compared to what local concentration these might have in the tissues themselves since mammary gland tumours are often surrounded by adipose tissue, administering the produced adipokines at close range, at concentrations much higher than those found in the serum.

We demonstrated that leptin and adiponectin exert opposing activities on the angiogenesis process, and that leptin is able to interact with various drugs clinically used in the management breast cancer.

Metastatic onset stems from the progression of the angiogenesis process and tumor invasion. Angiogenesis is a cascade of events that include endothelial cell proliferation, migration and endothelial tube formation. We studied each step of this process using HUVEC, which we found to be leptin and adiponectin receptor-positive, in accordance with previous reports showing that HUVEC express functionally-active leptin receptors [15], [30].

Our results clearly indicated that leptin was able to increase the proliferation of HUVEC in a VEGF-deprived media but had no effect in a complete media containing VEGF, which suggested that leptin treatment was associated with proliferation and/or survival of endothelial cells. This effect was significant at pharmacological leptin concentrations (1,000 ng/mL) and at concentrations reflecting obesity (100 ng/mL). The proliferative effect of leptin has already been reported in vascular cell lines [44]–[46] such as HUVEC [47] or bovine endothelial cells [33]. Even though Brakenhielm showed that adiponectin dose-dependently decreased proliferation in bovine and porcine endothelial cells (Brakenhielm 2004), we have shown here for the first time, to the best of our knowledge that adiponectin inhibits HUVEC proliferation, with a maximal effect within normal physiological concentrations (10,000 ng/mL). These changes in the cell proliferation process may be mediated through the deregulation of global apoptosis and/or cell cycle. Here, we showed that both leptin and adiponectin were able to inhibit global apoptosis. These data are consistent with Kobayashi *et al.* who showed that adiponectin decreased apoptosis in a dose-dependent manner [48], but in disagreement with Bouloumie *et al.* who found no effect of leptin on apoptosis [30], but the method and the concentration of leptin used were different in our experimental design. Caspase activation was also decreased by high-dose leptin and adiponectin, thus confirming the observed inhibition of global apoptosis. Indeed, leptin and adiponectin have opposite effects on cell proliferation, these activities can be explained by a change of apoptosis but also by modification of cell cycle. So, the inhibition of cell proliferation induced by adiponectin can be due to cell cycle inhibition, that counterbalanced anti-apoptotic effect.

Using 2 different *in vitro* models of angiogenesis (i.e. wound healing method and monolayer-induced formation of capillary-like tubes in Matrigel®), we demonstrated that leptin-induced migration was associated with an enhanced capillary-like tube formation. This finding is consistent with previous works [15], [30], [47]. It has been also reported that leptin increased HUVEC migration in a Boyden chamber [31].

Conversely, we found that adiponectin inhibited endothelial migration and tube formation. Brakenhielm *et al.* showed that adiponectin abolished VEGF-induced migration [19], but it has also been reported that adiponectin promotes endothelial cell migration and differentiation into tube-like structures *in vitro* [49].

The secretion of angiogenic factors by mammary tumor cells may be a key actor in the angiogenesis process. Here, we showed that leptin was able to increase VEGF secretion by MCF7 and MDA-MB-231 breast cancer cell lines. We failed to find VEGF in HUVEC in presence of leptin or adiponectin, probably due to the fast rate of VEGF consumption by the endothelial cells, contrary to Park *et al.* who showed a dose-dependent increase of VEGF secretion by HUVEC after leptin treatment [47].

Metastasis is facilitated by the development of neovascularization but is also dependent on the invasiveness of the cancer cells, and this study showed that leptin was able to increase the invasiveness of ER-positive MCF7 breast cancer cells but not ER-negative MDA-MB-231 cells, which are now well known to not respond to leptin treatment.

Another hypothesis to explain the higher breast cancer mortality rates in obese patients could be a potential decrease in treatment efficacy in this patient group. Tamoxifen (Tx), 5-fluorouracil (5-FU), taxol and vinblastin are important drugs for breast cancer treatment, and they all significantly inhibit cell growth. For the first time, our study showed that leptin decreased the anti-proliferative effect of Tx, 5-FU, taxol and vinblastin on MCF7 cells. These are vital findings, as it has previously been shown that anti-estrogen therapy, including Tx, can cause an increase in leptin secretion from adipocytes [38], [39]. However, the effects of leptin appear dependent on the cells studied, since Mełeń-Mucha *et al*. showed *in vitro* that leptin could increase the cytotoxic effect of 5-FU in colon cancer cells [41].

To the best of our knowledge, this is the first study to simultaneously compare the effects of leptin and adiponectin at concentrations comparable to physiological serum levels of normal weight or overweight/obese people on the angiogenesis process. Our data point to the conclusion that leptin and adiponectin may have opposite effects in the regulation of this process. The quiescent vasculature may represent a net balance between the production levels of angiogenic factors and inhibitors, where angiogenic factor overproduction or inhibitor underproduction would result in neovascularization and expansion. In obesity settings, where hyperleptinemia and hypoadiponectinemia were described, the pro-angiogenic effect of leptin was increased while the anti-angiogenic effect of adiponectin was decreased, thus suggesting the promoting formation of new vessels and the dissemination of tumor cells.

Moreover, leptin at high doses appears to reduce the effectiveness of different cancer treatments. Although leptin and adiponectin are shown to have contradictory effects, it is important to look at the wider picture, where adipocyte microenvironment appears to be a key direction for study. Further studies are therefore needed to better understand the role played by adipokines and adipocyte tumor microenvironment in breast cancer progression.
